# Associations between Barriers to Food Pantry Use, Visit Frequency, Pantry Experiences, and Amount of Food Received

**DOI:** 10.3390/nu16193334

**Published:** 2024-10-01

**Authors:** Haisu Zhao, Francine Overcash, Abby Gold, Marla Reicks

**Affiliations:** Department of Food Science and Nutrition, University of Minnesota, St. Paul, MN 55108, USA; zhao1930@umn.edu (H.Z.); overc006@umn.edu (F.O.); agold@umn.edu (A.G.)

**Keywords:** food pantry, barriers, visit frequency, 2022 Minnesota Food Shelf Survey

## Abstract

**Background/Objectives:** Barriers to food pantry use have been identified but little information is available regarding how these barriers are related to food pantry use. The purpose of this study was to assess relationships between barriers and (1) user demographic characteristics, (2) visit frequency and amount of food received, and (3) satisfaction with pantry visit experiences. **Methods:** Data were used from the 2022 Minnesota Food Shelf Survey, which included responses from 288 food pantries across the state with 6267 individuals reporting on barriers to pantry use. Survey barrier statements included limits on frequency of visits allowed, lack of reliable transportation, scheduling difficulties, and other. Survey satisfaction statements included being able to choose food types, being made welcome, having an easy selection process, having different varieties of food available, and foods having a favorable appearance. Chi-square and mixed model logistic regression analyses were used to assess relationships between reporting barriers and demographic characteristics, visit satisfaction, visit frequency, and amount of food received in the past 6 months. **Results:** Respondents were primarily non-Hispanic White (65%) and female (68%). Regression models showed that reporting barriers to pantry use was not associated with user demographic characteristics, but was associated with greater odds of visiting the pantry more often, and lower odds of getting more food from the pantry or reporting a satisfactory food pantry visit experience. **Conclusions:** Findings may be useful for food pantry staff to improve pantry access and visit experiences and for public health professionals who advise those who use food pantries to supplement household food supplies.

## 1. Introduction

In the United States, more than 20% of adults reported experiencing increased food insecurity post-pandemic with intensified use of food assistance programs [1]. In Minnesota, 7.5 million pantry visits were reported in 2023, which was almost twice the number reported in 2020, with nearly 200,000 children facing hunger [2,3,4]. However, perceived pantry barriers were reported in several studies [5,6,7,8,9,10], which may influence visit frequency and experiences and thus the ability to obtain food to meet family needs. 

Barriers to pantry use included limited transportation, associated stigma, insufficient information about the pantry, poor quality of foods available, dissatisfaction with the experience, clients’ complex medical conditions, limited pantry hours of operation, and lack of access to cultural foods [5,6,7,8,9,10]. In rural Minnesota, focus group participants also identified a barrier related to limited language choices available at food pantries [7]. Using logistic regression analysis, survey results from a Massachusetts statewide sample during the COVID-19 pandemic showed food pantry users in non-White communities (i.e., Black, Latinx) were more likely to report barriers than White adults [8]. Survey results in Northwest Arkansas showed that food pantry usage patterns were associated with client socioeconomic status, age, and medical conditions based on logistic regression analysis [9]. 

Several factors have been studied previously regarding user satisfaction with food pantry visits based on survey responses [8]. Long et al. [8] found that the majority of users reported positive food pantry experiences such as feeling welcome, receiving culturally acceptable foods, and having convenient hours, while a lower percentage of Latinx and Asian adults reported encountering pantry staff who spoke their language than White adults. Other factors addressing pantry user satisfaction were identified using qualitative research methods involving focus group and individual interviews with pantry users and staff [11,12,13,14,15]. A review of dimensions that contribute to user satisfaction with food pantry visits involving 50 food pantries in New York found that food availability was dependent on client position in line, having equipment for refrigerated and frozen foods, and having prepared foods available [11]. Limited pantry space and equipment were also identified by pantry coordinators in Cincinnati, Ohio, as factors that resulted in fewer food options for clients [12]. Similarly, interviews with pantry staff in St. Louis, Missouri, identified barriers to being able to provide healthy food options, which included limitations based on storage space, limited budget, and types of food donated [13]. Focus groups conducted in the Greater Bridgeport, Connecticut, area with food pantry patrons addressed continuous issues with access to healthy food, food specific to health problems, and getting enough food [14]. Patrons also mentioned disappointing pantry experiences based on long lines and how foods were distributed. Open-ended responses to the 2017 Minnesota statewide food pantry survey also showed concern over limited or low-quality food options, while other aspects of the pantry visit experience were positive including having positive interactions with staff in a safe and social environment [15]. The majority of studies regarding pantry user satisfaction were based on qualitative methods, while quantitative methods were not widely used to determine relationships between barriers to use and pantry user satisfaction.

Several studies have identified associations between frequency of food pantry use and diet quality and food security [16,17]. Based on Healthy Eating Index-2010 scores, Liu et al. [16] found that food pantry clients visiting food pantries more than once per month had better diet quality than those visiting less often. In addition, data from households in Dallas County, Texas, showed that an increased frequency of food pantry visits was associated with improvements in self-rated health and food security [17]. Increased visit frequency could potentially be related to receiving a greater amount of food over time, which may contribute to better diet quality and food security. However, studies of associations between barriers to pantry use and frequency of food pantry visits and amount of food received are limited.

While a variety of barriers to pantry use have been identified previously [5,6,7,8,9,10] and associations between barriers to use and pantry user characteristics have been reported to some extent [8], quantitative studies regarding associations between barriers to use and pantry user experiences, and behaviors regarding pantry use, are limited. A better understanding of how barriers to pantry use influence user satisfaction, visit frequency, and amount of food received may enhance the ability of pantries to provide food to meet the needs of families having food and nutrition insecurity. To address this research gap, the purpose of this study was to determine whether reporting barriers to use was associated with (1) demographic characteristics of users, (2) frequency of pantry visits and amount of food accessed, and (3) perceived satisfaction with experiences visiting the food pantry using quantitative data collection and analysis methods. We hypothesized that reporting barriers to pantry use would be associated with demographic characteristics of users, limited frequency of pantry visits and a smaller amount of food accessed, and with a less satisfactory food pantry visit experience. Findings may be used by pantry managers and staff to address policies related to pantry visit frequency and experiences. Results may also be useful for public health professionals in education and intervention efforts to improve food pantry experiences and benefits. 

## 2. Materials and Methods

### 2.1. Sample

The 2022 statewide Minnesota Food Shelf Survey was conducted from July to October 2022 through a collaboration of hunger relief organizations (food pantries, community charitable organizations, and state programs). Survey details and the questionnaire can be found online [18]. A pilot study was implemented in three food pantries to test the readability of questions assessing user perceptions of experiences and item availability, and revised as needed. The survey was available in five languages (English, Spanish, Somali, Hmong, and Russian). Food pantries distributed flyers with information about the survey on paper at food pantries and through emails and text messages with QR codes to access the survey online. Surveys were completed in-person on paper or online at one of the 288 food pantries across the state, or completed at home. 

Completed paper surveys were either returned to food pantry sites or mailed to the study site for data entry. For completing the survey, participants were enrolled in a gift card drawing. The University of Minnesota Institutional Review Board determined that this study was not considered human subjects research. 

### 2.2. Measures

#### 2.2.1. Demographic Characteristics

Self-reported demographic characteristics were included in the survey by asking participants to check all that apply to the question “What is your race and or ethnic background?” with the following response options: Alaska Native, Native American, Asian (including Southeast Asian), Native Hawaiian or Pacific Islander, African (e.g., Somali, Ethiopian, Liberian, Eritrean, etc.), Black and African American, Hispanic or Latinx, White (Caucasian), prefer not to answer, and other (please describe). If respondents checked more than one response, their response was recoded as other. 

Responses to the question “What is your gender?” included female, male, transgender female, transgender male, nonbinary, prefer not to answer, and other (please describe) with instructions to check one option. Responses to the transgender, nonbinary, and other categories were combined as other.

Household size was assessed by asking “How many people do you provide food for, including yourself?” Rural-Urban Commuting Area (RUCA) codes were applied to categorize regions into urban (metropolitan and micropolitan) and rural (small towns and rural areas) [19]. Participant responses were assigned a site number based on the pantry where they completed the survey.

#### 2.2.2. Food Pantry Variables

The food pantry visit frequency and the amount of food received from the pantry were measured through questions: “How often do you visit this food shelf (pantry)?” and “How much of all the food you got in the last 6 months was from this food shelf?” with instructions to check one option. Visit frequency options were does not apply, this is my first visit, a few times a year, once a month, a few times a month, and once a week or more. Response options for the amount of food included I didn’t get any, less than half, about half, more than half, and all of my food. Perceived barriers were assessed by asking “What barriers keep you from accessing food from this food shelf as frequently as you would like?’’ with check all that apply response options of I have no barriers to accessing food, The food shelf limits how often I can access food, I have trouble accessing reliable transportation to access food, The food shelf hours do not work for my schedule, and other (please describe). The pantry experience was evaluated by the survey question “How often do you experience the following at this food shelf?” with frequency options of never, rarely, sometimes, often, and always for each of the following statements: I can choose my own food, Volunteers or staff greet me and make me feel welcome, The process to select my food is easy, Plenty of different varieties of food are available, and Food looks fresh & appealing. 

#### 2.2.3. Data Analysis 

From the total sample of 6984 participants, data were deleted for those who did not respond to the question about barriers to using the food pantry, those who indicated they had no barriers but checked a barrier option, and those who indicated they preferred not to answer this question resulting in an ending sample of 6267 participants. For those who checked the other (please describe) option, responses were recoded into one of the three listed barrier options (hours do not work for schedule, limits on how often can access food, and trouble accessing reliable transportation to access food. If the description included information about other barriers (health, distance, not aware), the response was coded as other. If the description did not include barrier information, the response was recoded as having no barriers to accessing food. 

SAS OnDemand for Academics (Version 9.4) was used for data analysis. Responses to all survey questions where participants indicated prefer not to answer were recoded as missing. Demographic variables were dichotomized where Non-Hispanic White, Caucasian = 0 and all other groups = 1, female = 0 and male and other = 1, number of people in the household provided food for ≤2 = 0 and >2 = 1, and urban = 0 and rural = 1. Pantry experience variables were dichotomized where never, rarely, sometimes and often = 0 and always = 1. Pantry visit frequency reported as once a week or more, a few times a month, or once a month was coded as 1, and a few times a year, or does not apply, this is my first visit was coded as 0. The amount of food received from the food shelf reported as less than half or didn’t get any was coded as 0, and getting about half, more than half, or all of my food was coded as 1. Frequencies and percentages for survey responses were calculated for all participants and by number of barriers (0 or 1+). 

In an initial bivariate analysis, chi-square tests were used to examine simple comparisons between number of barriers and demographic characteristics, pantry experience variables, and behaviors regarding pantry use. Similar studies used logistic regression modeling to determine relationships between barriers to pantry use and pantry user characteristics and pantry usage patterns [8,9]. Therefore, mixed model logistic regression analyses were then used to determine the probability of associations between the categorical dependent variables (demographic variables, pantry experience variables, frequency of pantry visits, and amount of food received from the pantry) and the independent variable (number of barriers). Models were adjusted for fixed effect covariates (race, gender, number of people provided food for, and region) with site included as a random intercept to account for clustering effects [20,21]. Results were considered significant if *p* < 0.05. 

## 3. Results

### 3.1. Demographic Characteristics by Reporting Barriers

The final analysis included 6267 food pantry users that resided in Minnesota. The majority (64.9%) identified as non-Hispanic White with an additional 9.8% as Hispanic or Latinx, 8.5% as Other, and 6.5% as Black, African American (Table 1). The majority also identified as female (68.2%) and living in urban areas (71.9%). In addition, about half of all participants reported that they provide food for two or fewer household members (52.7%) or for three or more household members (47.4%).

Chi-square analysis showed that the percentage of those with barriers was higher than those without barriers for most non-White groups (except for Hispanic or Latinx), while the percentage of those with barriers was lower than those without barriers for non-Hispanic White participants (60.1% vs. 67.6%). Chi-square analysis also showed that of those with barriers, 3.6% identified as transgender or nonbinary compared to those with no barriers (0.6%). For participants with barriers, the percentage of those residing in urban areas was higher based on chi-square analysis compared to those without barriers (77.6% vs. 68.6%) and the percentage of those providing food for two or fewer households members was lower than for those without barriers (20.7% vs. 26.2%). However, the adjusted random intercept regression model analyses did not show significant differences in the number of barriers to pantry use by gender, race and ethnicity, the number of household member(s) the number of people in the household that the respondent provided food for, or region (Table 1).

Less than half (44.8%) of all respondents reported visiting the food pantry once per month (Table 1). About one-fifth (19.1%) of those not reporting barriers indicated this was their first visit compared to only 10.3% of those reporting one or more barriers. Those not reporting barriers compared to those reporting 1 or more barriers were less likely to indicate they visited the food pantry a few times a month (10.1% vs. 14.5%) and to report visiting the pantry a few times a year (23.9% vs. 26.2%). 

About one-third (36.1%) of all participants reported getting less than half of their food from pantries (Table 1). One-third reported getting about half of their food (29.4%) and one-fifth reported getting more than half of their food from pantries (21.0%). Only 9.2% reported receiving all of their food from the food pantry. Among those reporting barriers, 39.0% reported getting less than half of their food from the pantry compared to the 34.4% who indicated no perceived barriers. Of those reporting barriers, 7.0% reported getting all their food from the pantry compared to 10.5% not reporting barriers. 

After adjusting for sociodemographic correlates and site ID as a random intercept, participants who reported any barriers to food pantry use had an increased odds (~49%) of visiting the food pantry more often (OR = 1.49, *p* < 0.0001) and lower odds (OR = 0.700, *p* < 0.0001) of getting more food from the food pantry compared to those who reported no barriers (Table 1).

### 3.2. Barriers to Using the Food Pantry and Satisfaction with Experiences

A majority (n = 3981, 60.9%) reported no barriers to accessing food from food pantries. Those reporting one or more barriers reported trouble accessing transportation (n = 863, 12.9%), food pantry hours not working with schedules (n = 554, 8.5%), food pantries limiting how often participants could visit (n = 863, 13.2%), and other (n = 297, 4.5%). 

Overall, chi-square analyses showed that food pantry clients who reported no barriers indicated having a better shopping experience compared to food pantry clients with perceived barriers (Table 2). Those without barriers compared to those with barriers reported always being able to choose their food (69.9% vs. 59.4%), feeling welcome by pantry staff and volunteers (86.7% vs. 73.0%), that food selection was easy (80.4% vs. 65.9%), that different varieties of food were available (63.0% vs. 46.9%), and that the food always looked fresh and appealing (59.2% vs. 44.3%). Consistent with chi-square analyses, after adjusting for sociodemographic correlates and site ID as a random intercept, participants who reported any barriers to food pantry use had lower odds (all ORs = 0.560–0.651, all *p*s < 0.0001) of reporting a satisfactory pantry experience compared to those who reported no barriers.

## 4. Discussion

Overall, based on our hypotheses, we found that reporting barriers to pantry use was not associated with demographic characteristics of users, but was associated with increased odds of visiting the food pantry more often, and lower odds of getting more food from the pantry or reporting a satisfactory food pantry visit experience. Disparities in reporting barriers to pantry use were observed by race and gender based on simple comparisons; however, adjusted mixed model logistic regression analysis did not show significant effects, in contrast to our hypotheses and findings from other studies [8,14,22]. In Minneapolis/St. Paul, Minnesota, Caspi et al. found that areas with the highest concentration of minority groups had greater poverty rates but were also close to established food pantries [22]. Among Massachusetts residents, another study showed that Latinx and Black communities were less likely to secure food from food pantries compared to predominantly White communities because of limited transportation, inconvenient open hours, and pantry locations [8]. In addition, Russomanno and Jabson Tree found there was low prevalence of reporting pantry use among transgender or gender nonconforming people due to gender identity, social stigma, and minority stress [23]. Differences in how data were collected and analyzed between studies may partially explain why the results in the current study were not consistent with other findings.

Contrary to findings of others [16,24], the current study did not find that living in a small town or rural area was related to reporting barriers to pantry use. In a study of six rural counties, Byker Shanks et al. showed that households experienced hardships securing food [24]. A comparable study conducted in Indiana showed a higher frequency of reporting barriers in transportation and stigma in rural vs. urban regions [25]. In Minnesota, the SuperShelf Program, which is an intervention that provides pantry clients the open ability to choose food, has been implemented in many pantries across the state [26]. Therefore, the involvement of SuperShelf and other innovations, such as the use of mobile pantries, may have alleviated some of the burden for rural households to access food from pantries. 

In the current study, respondents reporting that they had at least one barrier also reported being less satisfied with the pantry experience (easy food selection, available varieties of food, fresh and appealing food, welcoming pantry staff, and food that fits personal preferences) than respondents without barriers. However, half of the survey respondents overall reported good experiences and services during pantry visits regardless of whether they reported barriers. In contrast to these study results, interviews conducted in Arkansas showed that pantry clients were in need of food that was higher quality (foods that did not expire, go rotten, or spoil) and in larger quantities, especially dairy and meat products [27]. From the 2017 Minnesota Statewide Food Pantry Survey, Caspi et al. found healthy food options were often desired by pantry clients but inconsistently available [28].

Individuals who reported having barriers to food pantry use in the current study were likely to receive less food from the pantry but have more pantry visits than those not reporting barriers. In contrast, a high food pantry usage pattern among pantry clients in Northwest Arkansas showed that those with the highest usage often received the most food from pantries [9]. Given the lower odds of getting greater amounts of food from food pantries by households with barriers in the current study, these respondents may have visited more often to mitigate food insecurity or tradeoffs between other non-food household needs. Households not reporting barriers may have been more capable of securing food from other sources rather than depending on emergency food systems; therefore, visit frequency could have been reduced for these households, which may have resulted in receiving more food per visit.

A strength of this study is its large statewide sample; however, the survey results lacked generalizability to other states with different major demographic characteristics. Another strength was having two survey formats available in paper and online so that households without either digital access or internet skills could access the survey. However, a large number of respondents did not answer some demographic questions, therefore the amount of missing data is a limitation of the analysis. Only three barrier options (the food shelf limits how often I can access food, I have trouble accessing reliable transportation to access food, and the food shelf hours do not work for my schedule) were included in the survey. Inclusion of other options such as physical/health conditions, and being unhoused would have allowed for more specificity.

## 5. Conclusions

Compared with households not reporting barriers in the current study, households reporting barriers had increased odds of visiting the food pantry more often, and lower odds of getting more food from the pantry or reporting a satisfactory food pantry visit experience. The findings provide insights for pantry managers and staff and public health professionals to educate and assist pantry users facing barriers to food access to improve the amount of food received and satisfaction with the pantry experience. The data also indicated that pantry managers and staff could improve the pantry visit experience by revising pantry policies based on additional study. For example, studies could be conducted to assess how expanding hours the pantry is open and revising the limits on the amount of food that can be accessed would influence food security and diet quality of pantry users as well as feasibility of these changes by pantry managers and staff in line with capacity and budget considerations. Findings from the current study also support the need for further longitudinal surveys to assess how policy changes affect outcomes among pantry users and pantry organizations. 

## Figures and Tables

**Table 1:** Demographic and other characteristics of respondents by reporting barriers to food pantry use (none vs. 1+ barriers).

Characteristic	All n (%) n = 6267	No Barriers Reported n (%) n = 3981	1+ Barriers Reported n (%) n = 2286	Chi-Square *p* Value ^1^	Odds Ratio	CI ^2^	Regression Model *p*-Value ^3^
Gender (n = 718 missing)				<0.0001	1.022	0.858, 1.217	0.8099
Male	1672 (30.1)	1121 (31.7)	551 (27.4)				
Female	3785 (68.2)	2395 (67.7)	1390 (69.1)				
Other (transgender female or male, nonbinary, other)	92 (1.7)	20 (0.6)	72 (3.6)				
Race and ethnicity (n = 913 missing)				<0.0001	0.969	0.804, 1.168	0.7396
Hispanic or Latinx	522 (9.8)	337 (9.9)	185 (9.5)				
Non-Hispanic White, Caucasian	3472 (64.9)	2299 (67.6)	1173 (60.1)				
Non-Hispanic Black, African American	346 (6.5)	207 (6.1)	139 (7.1)				
Non-Hispanic Asian (Southeast Asian, Native Hawaiian or Pacific Islander)	170 (3.2)	99 (2.9)	71 (3.6)				
Alaska Native, Native American	279 (5.2)	139 (4.1)	140 (7.2)				
African	111 (2.1)	56 (1.7)	55 (2.8)				
Other race-including multiracial	454 (8.5)	266 (7.8)	188 (9.6)				
Number of household member(s) respondent provides food for (n = 1206 missing)				<0.0001	1.099	0.926, 1.304	0.2786
<2	1435 (28.4)	943 (28.7)	492 (27.7)				
2	1228 (24.3)	860 (26.2)	368 (20.7)				
3–4	1296 (25.6)	815 (24.8)	481 (27.1)				
5–16	1102 (21.8)	667 (20.3)	435 (24.5)				
Region (n = 15 missing)				<0.0001	0.773	0.205, 1.963	0.5883
Urban (metropolitan + micropolitan)	4494 (71.9)	2725 (68.6)	1769 (77.6)				
Rural (small town + rural)	1758 (28.1)	1246 (31.4)	512 (22.5)				
How often do you visit this food shelf? (n = 294 missing)				<0.0001	1.485	1.218, 1.807	<0.0001
Does not apply, this is my first visit	947 (15.9)	721 (19.1)	226 (10.3)				
A few times a year	1477 (24.7)	904 (23.9)	573 (26.2)				
Once a month	2674 (44.8)	1678 (44.4)	996 (45.5)				
A few times a month	700 (11.7)	383 (10.1)	317 (14.5)				
Once a week or more	175 (2.9)	96 (2.5)	79 (3.6)				
How much of the food you got in the last 6 months was from this food shelf? (n = 1259 missing)				<0.0001	0.700	0.596, 0.819	<0.0001
I didn’t get any	237 (4.3)	136 (3.9)	101 (5.1)				
<half	1990 (36.1)	1212 (34.4)	778 (39.0)				
About half	1620 (29.4)	1053 (29.9)	567 (28.4)				
>half	1161 (21.0)	750 (21.3)	411 (20.6)				
All of my food	509 (9.2)	369 (10.5)	140 (7.0)				

^1^ *p*-value based on chi-square tests, ^2^ CI = confidence interval, ^3^ *p*-value based on adjusted random intercept regression models.

**Table 2:** Respondents report of food pantry visit experience by reporting barriers to food pantry use (none vs. 1+ barriers).

Experience	All n (%) n = 6267	No Barriers Reported n (%) n = 3981	1+ Barriers Reported n (%) n = 2286	Chi-Square *p* Value ^1^	Odds Ratio	CI ^2^	Regression Model *p*-Value ^3^
I can choose my own food. (n = 518 missing)				<0.0001	0.651	0.544, 0.780	<0.0001
Never	396 (6.9)	245 (6.7)	151 (7.2)				
Rarely	178 (3.1)	88 (2.4)	90 (4.3)				
Sometimes	516 (9.0)	259 (7.1)	257 (12.3)				
Often	860 (15.0)	512 (14.0)	348 (16.7)				
Always	3799 (66.1)	2559 (69.9)	1240 (59.4)				
Volunteer or staff greet me and make me feel welcome. (n = 845 missing)				<0.0001	0.577	0.480, 0.694	<0.0001
Never	48 (0.8)	27 (0.7)	21 (1.0)				
Rarely	40 (0.7)	13 (0.4)	27 (1.3)				
Sometimes	365 (6.5)	156 (4.4)	209 (10.3)				
Often	766 (13.7)	397 (11.2)	369 (18.1)				
Always	4203 (75.1)	2862 (80.4)	1341 (65.9)				
The process to select my food is easy (n = 673 missing)				<0.0001	0.560	0.468, 0.671	<0.0001
Never	177 (3.2)	104 (2.9)	73 (3.6)				
Rarely	83 (1.5)	40 (1.1)	43 (2.1)				
Sometimes	365 (6.5)	156 (4.4)	209 (10.3)				
Often	766 (13.7)	397 (11.2)	369 (18.1)				
Always	4203 (75.1)	2862 (80.4)	1341 (65.9)				
Plenty of different varieties of food are available. (n = 580 missing)				<0.0001	0.564	0.479, 0.665	<0.0001
Never	60 (1.1)	25 (0.7)	35 (1.7)				
Rarely	133 (2.3)	60 (1.7)	73 (3.6)				
Sometimes	880 (15.5)	446 (12.3)	434 (21.1)				
Often	1362 (24.0)	811 (22.4)	551 (26.8)				
Always	3252 (57.2)	2286 (63.0)	966 (46.9)				
Food looks fresh and appealing (n = 609 missing)				<0.0001	0.612	0.520, 0.721	<0.0001
Never	38 (0.7)	17 (0.5)	21 (1.0)				
Rarely	71 (1.3)	26 (0.7)	45 (2.2)				
Sometimes	849 (15.0)	432 (12.0)	417 (20.2)				
Often	1659 (29.3)	911 (27.6)	668 (32.3)				
Always	3041 (53.8)	2125 (59.2)	916 (44.3)				

^1^ *p*-value based on chi-square tests, ^2^ CI = confidence interval, ^3^ *p*-value based on adjusted random intercept regression models.

## Data Availability

The original contributions presented in the study are included in the article, further inquiries can be directed to the corresponding author.
